# Precision medicine: Sustained response to erdafitinib in *FGFR2* ‐mutant, multiply recurrent ameloblastoma

**DOI:** 10.1002/cnr2.1656

**Published:** 2022-06-27

**Authors:** Katherine A. Lawson‐Michod, Christopher H. Le, Ghassan Tranesh, Penelope C. Thomas, Julie E. Bauman

**Affiliations:** ^1^ UA Health Sciences University of Arizona (UA) Cancer Center Tucson Arizona USA; ^2^ Huntsman Cancer Institute University of Utah Health Salt Lake City Utah USA; ^3^ Department of Otolaryngology – Head & Neck Surgery UA College of Medicine‐Tucson Tucson Arizona USA; ^4^ Department of Pathology UA College of Medicine‐Tucson Tucson Arizona USA; ^5^ Department of Medical Imaging UA College of Medicine‐Tucson Tucson Arizona USA; ^6^ Department of Medicine UA College of Medicine‐Tucson Tucson Arizona USA; ^7^ Division of Hematology/Oncology, Department of Medicine George Washington (GW) University and GW Cancer Center Washington District of Columbia USA

**Keywords:** ameloblastoma, erdafitinib, FGFR2 mutation, precision medicine, targeted therapy

## Abstract

**Background:**

Ameloblastoma imposes significant morbidity and high‐recurrence rates following surgery and radiation therapy. Although 89% of cases harbor oncogenic mutations, the role of targeted therapy is undefined.

**Case:**

We describe a case of a 40‐year‐old male with multiply recurrent, locally invasive ameloblastoma of the posterior maxillary ridge. The tumor was unresectable for negative margins due to extensive intracranial disease, and the patient suffered severe symptoms including pain. Immune and genomic profiling were obtained to guide systemic treatment, showing a PD‐L1 score of 2% and *FGFR2* ^*V395D*^ and *SMO* ^*W535L*^ mutations. The patient progressed rapidly on anti‐PD1 immunotherapy. He was treated with the FGFR inhibitor, erdafitinib, with excellent partial response including resolution of intracranial disease and cancer‐related pain, ongoing 2 years after drug initiation.

**Conclusion:**

Targeting the *FGFR2* mutation resulted in sustained response and improved quality of life. Genomic profiling with targeted therapy for ameloblastoma appears promising, especially when surgery is technically infeasible.

## 1 INTRODUCTION

Ameloblastoma is a benign, locally aggressive, odontogenic tumor with a high‐recurrence rate and potential for malignant transformation and metastasis.^1^ Standard treatments include surgery and radiation therapy.^2^ Effective systemic therapy for ameloblastoma is poorly defined, however, recent identification of recurrent activating mutations in the mitogen activating protein kinase (MAPK) and Hedgehog pathways provides rationale for targeted molecular therapies.^2^, ^3^ Fibroblastic growth factor receptor 2 (*FGFR2*) mutations are identified in 6% of ameloblastomas.^3^ Erdafitinib is an oral FGFR inhibitor indicated by the U.S. Federal Drug Administration in *FGFR*‐mutant urothelial carcinomas.^4^ Clinical activity also has been reported in the off‐label treatment of *FGFR*‐mutant advanced cholangiocarcinoma^5^ and pancreatic adenocarcinoma.^6^ Here, we present a case of locally advanced, multiply recurrent *FGFR*‐mutant ameloblastoma treated with erdafitinib with prolonged partial response.

## 2 CASE

**FIGURE 1:**
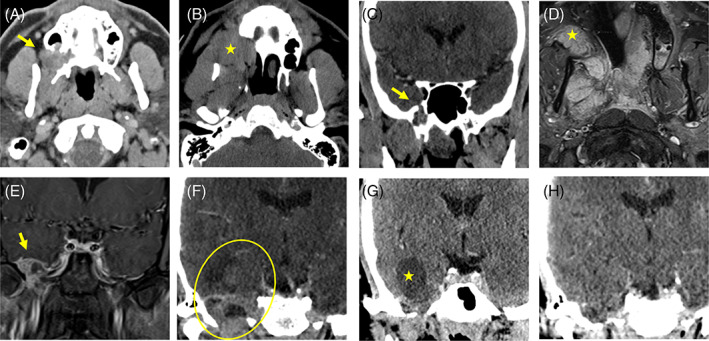
Radiologic findings throughout the disease course. *First recurrence (2013)*: Axial contrast‐enhanced CT (A) demonstrates part solid and cystic mass (arrow) centered in the right first maxillary molar alveolus with bony erosion and extension into the maxillary sinus. *Second recurrence (2016)*: Axial contrast‐enhanced CT (B) shows recurrent mass with increased involvement of the right hard palate and extension into right pterygopalatine fossa and masticator space. *Third recurrence (2018)*: Coronal contrast‐enhanced CT images (C) show increased infratemporal fossa involvement with expansion of right foramen rotundum and suspected early intracranial extension into middle cranial fossa (arrow). *Pre‐pembrolizumab baseline (2019)*: Axial (D) and coronal (E) contrast‐enhanced T1 weighted magnetic resonance imaging now 1.5 years after skull base resection, right total maxillectomy, and septoplasty shows extensive multifocal recurrence with expansile, enhancing masses in the right middle cranial fossa (arrow), masticator space, and submucosal buccal space (star). *Progression on pembrolizumab (2019)*: Off‐coronal contrast‐enhanced CT (F) after four cycles of pembrolizumab demonstrates progression, with enlarging solid and cystic intracranial tumor in the right middle cranial fossa (oval). *Response to erdafitinib (2020)*: Off‐coronal contrast‐enhanced CT 1 month (G) and 4 months (H) after start of erdafitinib show initial decrease in solid component with persistent cyst in the right middle cranial fossa (star), followed by marked decrease in intracranial component of tumor.

**FIGURE 2:**
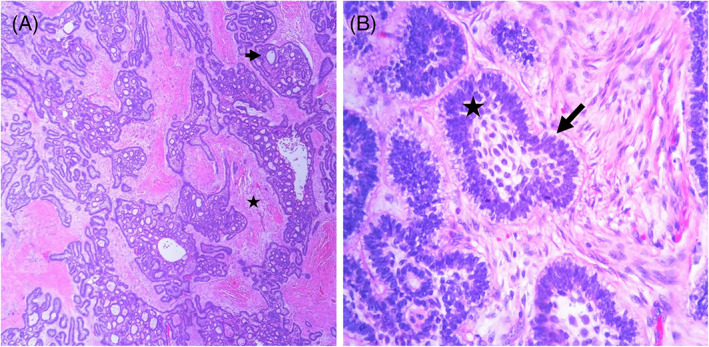
Histology of ameloblastoma at third recurrence. (A) Low power shows follicular growth pattern with islands of odontogenic epithelium (arrow) with peripheral palisading surrounding the fibrous stroma (star). (B) High power shows the central stellate reticulum (arrow) surrounded by palisaded columnar cells with reverse polarization (star).

A 40‐year‐old Caucasian male presented with a 10‐year history of multiply recurrent, locally invasive, life‐threatening ameloblastoma of the right maxillary sinus. The two initial surgeries had positive margins and included marsupialization of a maxillary sinus cyst with recurrence 2 years later (Figure 1A) and subsequent partial maxillectomy. Three years later, he was found to have recurrence in the maxillary sinus with extension into the pterygopalatine and infratemporal fossae (Figure 1B). He underwent a right total maxillectomy with resection of the skull base disease with negative margins. Pathology demonstrated conventional ameloblastoma. His surveillance exam and imaging 1 year after surgery showed no evidence of disease. Six months later, the patient developed right temporal pain and trismus. Cross‐sectional imaging showed multicentric recurrence in the right infratemporal fossa with extension into the lateral orbit, masticator space, and middle cranial fossa with invasion of the temporal lobe (Figure 1C–E). Endoscopic biopsies from the right pterygopalatine fossa, skull base, and maxilla were consistent with conventional ameloblastoma without malignant transformation (Figure 2), confirming the third recurrence. Gross total resection was not possible due to extensive intracranial extension. The patient refused definitive or palliative radiation therapy. He was referred to medical oncology where tumor immune and genomic profiling using the FoundationOne CDx (Cambridge, MA, USA) revealed a PD‐L1 combined positive score (CPS) of 2% by the 22c3 antibody as well as *FGFR2* ^*V395D*^ and smoothened homolog precursor (*SMO*)^*W535L*^ mutations. The patient was treated with the anti‐PD1 immune checkpoint inhibitor, pembrolizumab, however progressed after 12 weeks with increasing proptosis, trismus, and pain (Figure 1F). Due to *FGFR2* mutation, the small molecule FGFR inhibitor erdafitinib was started at 8 mg daily. Within 4 weeks, the patient observed a decrease in right maxillary fullness, periorbital edema, and cancer‐related pain such that he discontinued opioids. Computed tomography (CT) 1 and 4 months after erdafitinib initiation showed an excellent partial response (Figure 1G,H), with essential resolution of intracranial disease. Erdafitinib side effects included hyperphosphatemia and dry eyes, which resolved with routine use of calcium carbonate and ocular lubricants. After 12 months, grade 2 myalgias and fatigue prompted a treatment holiday. The disease remained in radiologic partial response without treatment for 14 months, when the patient developed multicentric progression associated with recurrent pain, trismus, and proptosis. Erdafitinib was restarted at 8 mg daily; clinical response was again observed after 4 weeks with improvement in pain, and CT assessment demonstrated repeat near‐complete response at 12 weeks.

## 3 DISCUSSION

Ameloblastoma most commonly presents as a locally invasive tumor with 70 percent of tumors undergoing malignant transformation and 2% metastasizing.^7^ Currently, the World Health Organization classifies ameloblastoma as benign while recognizing two malignant forms, metastatic ameloblastoma and ameloblastic carcinoma.^7^, ^8^ Although classified as benign, ameloblastoma can have severe clinical consequences. Following wide local excision, the current standard of care, patients experience significant morbidity and high recurrence rates.

Balancing quality of life and survival is a distinct challenge in managing ameloblastoma. Wide surgical resection is essential given high recurrence rates, however, is often morbid.^2^, ^7^ Radiation therapy can mitigate morbidity and improve progression‐free survival. Insights into the etiopathogenesis of ameloblastoma provide theoretical evidence for personalized, molecular targeted therapy. Specifically, genomic sequencing identified recurrent somatic, activating, mutually exclusive mutations in the MAPK pathway in 74 of 84 cases (88%) of ameloblastoma, including 62% *BRAF* ^*V600E*^, 20% *RAS*, and 6% *FGFR2*.^3^ In addition, recurrent, non‐exclusive mutations were observed in *SMO*, an oncogene in the Hedgehog pathway, *CTNNB1*, *PIK3CA*, and *SMARCB1*. The MAPK and Hedgehog pathways are involved in tooth morphogenesis.^3^, ^6^ *SMO* mutations have been associated with higher recurrence rates.^7^ Multiple reports of complete or prolonged partial responses following treatment with BRAF inhibitors in *BRAF*‐mutant ameloblastoma have shown the potential clinical impact of applying precision medicine.^9^, ^10^, ^11^

To our knowledge, this is the first reported case of ameloblastoma treated with erdafitinib. This patient's sustained response and improved quality of life suggest the utility of erdafitinib in *FGFR*‐mutant ameloblastoma, especially where surgery is technically infeasible or would pose severe morbidity. Indeed, approximately 89% of cases of ameloblastoma harbor at least one oncogenic mutation with an available inhibitor indicated in a different malignancy, including vemurafenib and dabrafenib (*BRAF*), erdafitinib (*FGFR*), alpelisib (*PIK3CA*), and vismodegib (*SMO*), raising the promise for personalized systemic therapy in morbid and malignant cases.

## AUTHOR CONTRIBUTIONS

**Katherine Lawson‐Michod:** Writing – original draft (equal); writing – review and editing (equal). **Christopher Le:** Writing – review and editing (equal). **Ghassan Tranesh:** Visualization (equal); writing – review and editing (equal). **Penelope Thomas:** Visualization (equal); writing – review and editing (equal). **Julie E. Bauman:** Writing – original draft (equal); writing – review and editing (equal).

## CONFLICT OF INTEREST

The authors have stated explicitly that there are no conflicts of interest in connection with this article.

## ETHICS STATEMENT

Written informed consent was obtained from the patient for publication of case details, including imaging.

## Data Availability

The data underlying this case report are not available for sharing due to privacy and ethical restrictions.
